# Nata de Cassava Type of Bacterial Cellulose Doped with Phosphoric Acid as a Proton Exchange Membrane

**DOI:** 10.3390/membranes13010043

**Published:** 2022-12-29

**Authors:** Andarany Kartika Sari, Rozan Mohamad Yunus, Edy Herianto Majlan, Kee Shyuan Loh, Wai Yin Wong, Nur Ubaidah Saidin, Sagir Alva, Deni Shidqi Khaerudini

**Affiliations:** 1 Fuel Cell Institute, Universiti Kebangsaan Malaysia, Bangi 43600, Selangor, Malaysia; 2 Engineering Faculty, Universitas Mercu Buana, South Meruya No. 1 Kembangan, West Jakarta 11650, Indonesia; 3 Research Center for Advanced Materials, National Research and Innovation Agency (BRIN), Kawasan Puspitek Serpong, South Tangerang 15314, Indonesia

**Keywords:** bacteria cellulose, nanocomposite membrane, conducting membrane, energy application, fuel cell

## Abstract

This work aims to encourage the use of natural materials for advanced energy applications, such as proton exchange membranes in fuel cells. Herein, a new conductive membrane produced from cassava liquid waste was used to overcome environmental pollution and the global crisis of energy. The membrane was phosphorylated through a microwave-assisted method with different phosphoric acid, (H_3_PO_4_) concentrations (10–60 mmol). Scanning electron microscopy (SEM), X-ray diffraction analysis (XRD), dynamic mechanical analysis (DMA), swelling behavior test, and contact angle measurement were carried out on the membrane doped with different H_3_PO_4_ levels. The phosphorylated NdC (nata de cassava) membrane doped with 20 mmol (NdC20) H_3_PO_4_ was successfully modified and significantly achieved proton conductivity (maximum conductivity up to 7.9 × 10^−2^ S cm^−1^ at 80 °C). In addition, the fabricated MEA was assembled using an NdC20 membrane with 60 wt% Pt/C loading of 0.5 mg cm^−2^ for the anode and cathode. Results revealed that a high power density of 25 mW cm^−2^ was obtained at 40 °C operating temperature for a single-cell performance test. Thus, this membrane has the potential to be used as a proton exchange membrane because it is environment-friendly and inexpensive for fuel cell applications.

## 1. Introduction

Biopolymers are the primary components of natural materials, and cellulose is one of the most abundant and common organic carbohydrate polymer materials on Earth [1] and has a promising role in the structural integrity and function of plants [2]. Cellulose is an unlimited raw material and a major source of environment-friendly materials for various applications, such as food, medical [3], energy storage, and electronic industries [4]. In addition, the development of cellulose as a new conducting membrane with good flexibility and dimensional stability has received great attention in recent years. This is because of growing environmental awareness and innovative uses of green technology to solve global challenges such as population expansion, energy crisis, and pollution [5].

Organic polymers have inherent advantages over synthetic polymers, such as low cost, biodegradability, and abundance [6]. Researchers and industrial producers are interested in cellulose derived from plants or bacteria because it is a sustainable green resource that is also renewable, degradable, biocompatible, and cost-effective. A.J Brown, in 1886, first reported that cellulose can be produced by microorganisms and was particularly popular in his country under the name of “vinegar plant” [7]. Herein, cellulose is obtained by the activity of microorganisms through different bacteria, processes, cultures, etc. [8]. Cellulose can be produced by *Acetobacter*, *Rhizobium*, *Agrobacterium*, *Aerobacter*, *Achromobacter*, *Azotobacter*, *Salmonella*, *Escherichia*, and *Sarcina* [9,10]. The best-known and most studied *Acetobacter xylinum* has been used for BC fabrication [11].

A proton exchange membrane should have high conductivity, good thermal stability, good absorbency, and low production cost. Bacterial cellulose (BC) is an interesting renewable green nanomaterial characterized by unique and advantageous properties, such as excellent mechanical strength, thermal stability, cost-effectiveness, wide availability, biodegradability, biocompatibility, water absorbance, and high crystallinity. BC is a very promising green material for various fields, such as food, biomedical [12], electronic, and energy applications [6,7,8]. BC has unique physical and chemical properties, such as a higher crystallinity index (above 60%), cross-sectional dimension in the nanometer range, hydrophilicity, and different degrees of polymerization (DP) between 2000 and 6000, compared with plant cellulose [5]. BC is also free of lignin, hemicelluloses, and pectin, which are found in plant-derived celluloses. In particular, the hemicellulose component is highly hydrophilic and has a high swelling ability, permeability, porosity, and water content [11], leading to a good flow of fluid across the membrane and high selectivity for mass transport of plant cellulose marked only 40%–70% of cellulose and required purification; however, purification of plant cellulose typically requires harsh chemicals [5,10,11,12]. 

Pure BC has a deficiency in optical transparency, electrical conductivity, magnetism, hydrophobicity, and antimicrobial properties [13]. In this regard, its capabilities should be improved and modified for various applications, such as conducting materials [14,15,16] and electrical devices [9,16]. BC is not naturally conducting; as such, it should be first converted into electrically conductive composites by incorporating conductive materials, such as nano carbon as fillers [8,15,17], as well as doping with acid and conducting polymers [18,19] in the form of nanoparticles or nanowires. Several studies produced PEM by using BC. In 2017, Gadim et al. [20] produced proton-conducting electrolytes from BC composite with poly(4-styrene sulfonic acid) (PSSA) at room temperature, with a yield of 40 mW cm^−2^ at 125 mA cm^−2^. Naumi et al. in 2018 [21] developed a polymer electrolyte membrane fuel cell based on BC with sulfonated polystyrene and phosphoric acid, yielding the proton conductivity of 7.17 × 10^−3^; phosphoric acid could improve the proton conductivity of membranes. Nata de cassava is a type of BC produced from the fermented liquid waste of *cassava* with the help of bacteria. The conductivity is very low, and the membrane is added with phosphate groups to improve the proton conductivity, as the phosphate group could be a medium for proton carriers to increase conductivity. In this work, a new conducting membrane was produced from cassava liquid waste. It has high proton conductivity and low cost and, thus, could be an alternative proton exchange membrane. The present study aimed to determine the effect of phosphorylation on the ion exchange capacity, ionic conductivity, swelling index, and contact angle of the membrane. The power density performance of the membrane was also evaluated in real H_2_/O_2_ fuel cells at various temperatures (25–80 °C).

## 2. Materials and Methods

### 2.1. Materials

*Cassava* was purchased from a local market, and *A. xylinum* bacteria were obtained from Malaysian Agricultural Research and Development Institute (MARDI). Sucrose, acetic acid, phosphoric acid, sodium hydroxide (NaOH), and dimethyl formamide (DMF) were obtained from Fisher Scientific.

### 2.2. Synthesis of Phosphorylated Nata de Cassava Membrane (NdC)

**Figure 1:**
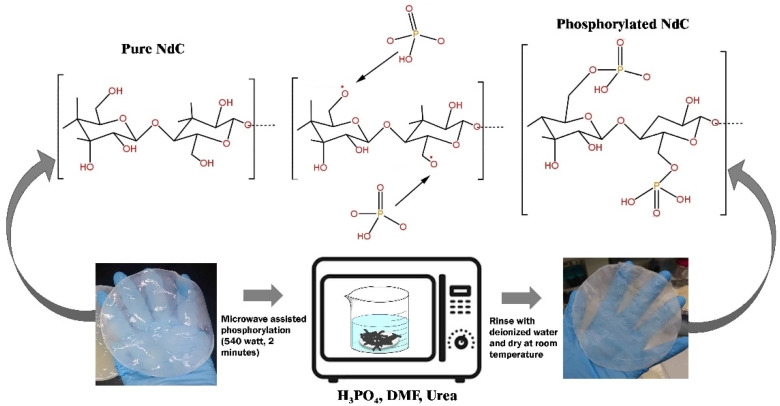
Phosphorylation process of Nata de Cassava.

NdC and phosphorylated NdC were prepared following the procedure in our previous work, and Figure 1 shows the phosphorylation process [22]. Microwave-assisted organic synthesis can be used to rapidly explore chemistry space and increase the diversity of compounds produced [23]. In the present study, microwave irradiation was used to synthesize phosphorylated NdC. The NdC membrane was purified and dried based on the previous method [24]. 

### 2.3. Morphology Characterization

Membrane surface morphology was evaluated using Field-Emission Scanning Electron Microscope (FESEM) Carl Zeiss/GeminiSEM 500 operating at 5 kV. The samples were previously coated with platinum and analyzed at a magnification of 10.00 K X.

### 2.4. XRD Analysis

X-ray diffraction was performed using a PANalytical X’Pert PRO using Cu Kα radiation (λ = 1.541 Å) with a scan of 0.05°s^−1^ on a 2θ scale to investigate the crystallinity of the NdC membranes.

### 2.5. Proton Conductivity

The membrane proton conductivity was measured using an electrochemical impedance analyzer (Autolab PGSTAT128N potentiostat). The frequency rate was 30 Hz to 2 MHz at 10 mV. Proton conductivity was measured on a 2 cm^2^ membrane placed in a Teflon cell containing two stainless steel electrodes in a controlled temperature chamber. Electrochemical impedance spectroscopy analysis was carried out to select the highest conductivity of the phosphorylated NdC. Proton conductivity was measured at room temperature up to 120 °C. Herein, proton conductivity was calculated by Equation (1) as follows:

(1) $$\sigma =\frac{t}{A\;\times \;R}$$

where:

σ = proton conductivity
t = membrane length
A = membrane surface area
R = ionic resistance

### 2.6. Ion Exchange Capacity (IEC)

IEC measurement is an important analytical technique for determining the activity of a substance undergoing an ion exchange process with other ions that exist in the environment. IEC was measured by titration. The dried phosphorylated NdC membrane was soaked in 50 mL of NaCl (1 M) solution for 24 h to release all the H^+^ ions out of the sample. The sample was then dried in an oven at 80 °C. The NaCl solution was titrated with NaOH solution (0.1 M) with a phenolphthalein as an indicator.

*IEC* value was calculated using Equation (2) as follows:

(2) $$IEC=\frac{\left(V_{b}-V_{s}\right)\left[\mathrm{Acid}\right]f_{p}}{m}$$

### 2.7. Contact Angle and Water Uptake

Contact angle analysis was performed at Research Center for Physics, Indonesian Institute of Science (LIPI) by using Dino-Lite digital microscope dremler AM3111/3113 series. The sample size in 2 cm^2^ water droplets (10 μL) was dropped on the membrane surface, and static contact angles were measured. The water uptake of the phosphorylated NdC membrane was determined according to a previous method [25] through swelling thickness. The membrane was weighed first under a dry condition, and its thickness was measured. The membrane was then soaked in water for 24 h at room temperature. The thickness of the membrane was measured, and its weight was recorded under a wet condition. Three samples were measured for each condition, and the average was taken.

Water uptake was calculated using Equation (3) as follows:

(3) $$\mathrm{Water}\;\mathrm{uptake}\;\left(\%\right)=\frac{\mathrm{Wwet}-\mathrm{Wdry}}{\mathrm{Wdry}}\times 100\%$$

### 2.8. Dynamic Mechanical Analysis (DMA)

The mechanical strength of the membranes was determined using a dynamic mechanical analyzer (PerkinElmer DMA 8000) at a frequency of 1 Hz and a heating rate of 3 °C/min from 25 °C to 250 °C.

### 2.9. Single-Cell Performance

PEMFC performance was tested using a fuel cell test station apparatus. The catalyst loading (60 wt% Pt/C) was set as 0.5 mg cm^−2^ for both electrodes. The anode and cathode were placed on both sides of the phosphorylated NdC before hot pressing at 80 °C and 30 bar for 3 min. Performance was evaluated in a 5 cm^2^ single cell from 25 °C to 80 °C. The cell was fed with H_2_ (0.08 L min^−1^) as fuel and O_2_ (0.2 L min^−1^) as an oxidant to evaluate the single-cell performance simultaneously.

## 3. Results

### 3.1. Morphology Characterization

**Figure 2:**
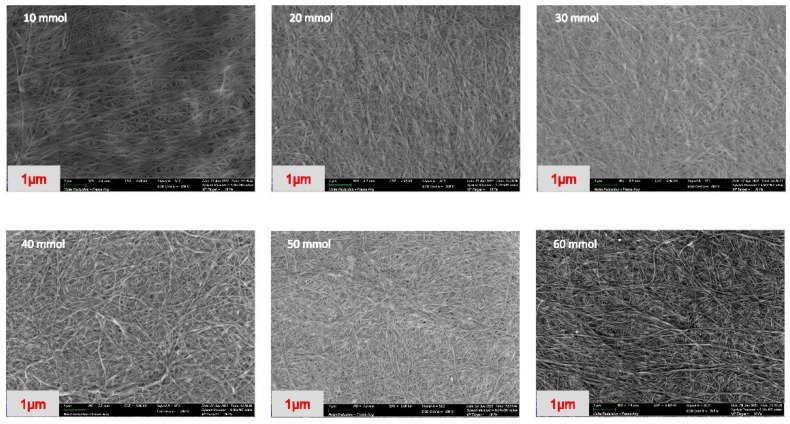
SEM morphology of phosphorylated NdC (10–60 mmol).

The BC membrane is a 3D network composed of abundant nanoscale fibers. The surface morphologies at various concentrations of the phosphorylated NdC were evaluated by field emission scanning electron microscopy (SEM). Figure 2 presents the FESEM images of the phosphorylated NdC with different concentrations of phosphoric acid. In general, pure BC has an average diameter of 40–100 nm and a length ranging from micrometers to dozens of micrometers.

Table 1 shows the diameter of the fiber, which consists of phosphorylated NdC with different phosphoric acid concentrations (10–60 mmol). The thickness of the cellulose fiber increased from 67.5 nm to 415.4 nm with increasing phosphoric acid concentration. The membrane preserved the porous structure of BC, which is expected to significantly improve the liquid electrolyte adsorption and support the ion transport through the 3D network channels [26]. 

### 3.2. XRD Analysis

**Figure 3:**
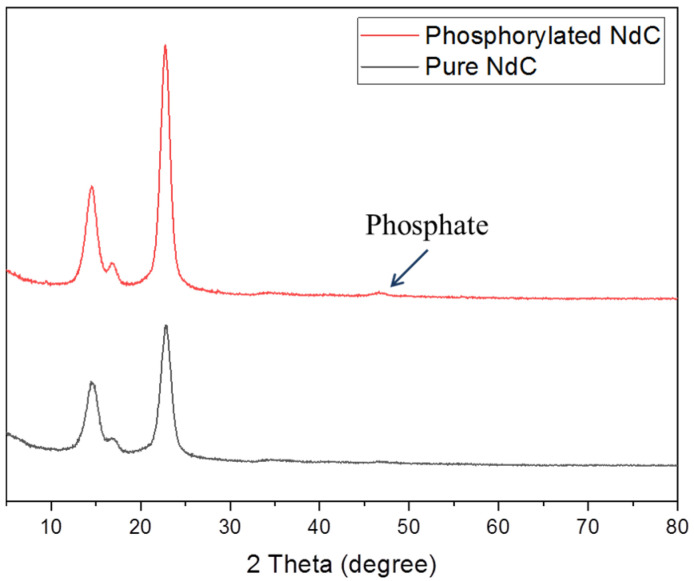
XRD spectra of pure NdC and phosphorylated NdC (20 mmol).

The X-ray diffraction patterns of the pure NdC and phosphorylated 20 mmol NdC are shown in Figure 3. Three distinct peaks were observed at 2θ = 14.6°, 16.7°, and 23.8°, which can be attributed to the (100), (110), and (200) reflections of cellulose peaks [27]. The phosphorylated NdC showed the existence of a phosphate peak at 47° and the presence of a crystalline region, thereby indicating the higher crystallinity of the membrane phosphorylated with phosphoric acid [28]. 

### 3.3. Ion Exchange Capacity, Proton Conductivity, and Contact Angle of Phosphorylated NdC

**Figure 4:**
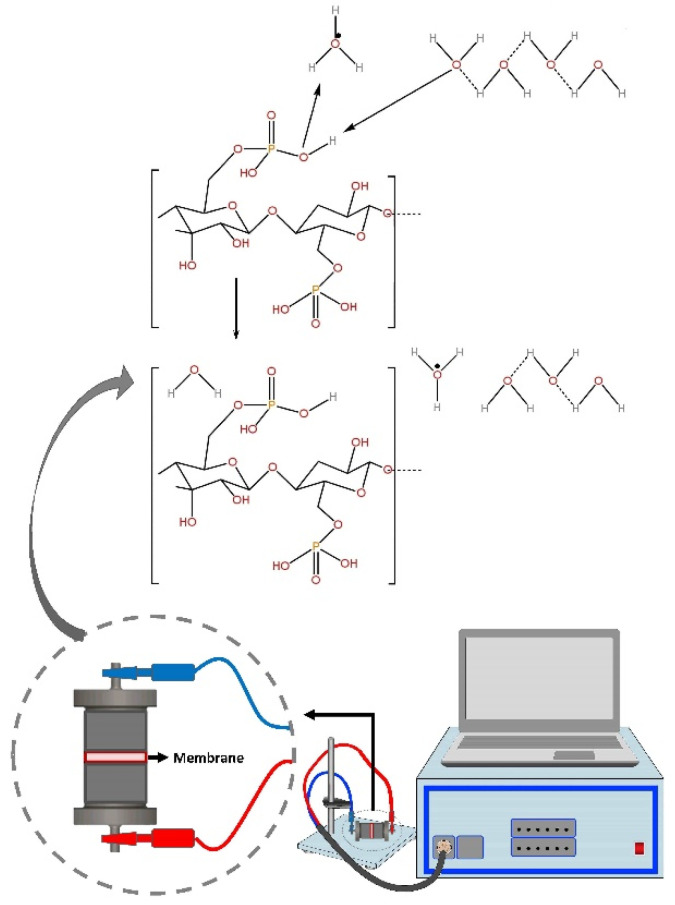
Schematic of ion transport mechanism of the phosphorylated NdC membrane.

The rate of proton transport in electrolytes is a crucial factor for the development of conducting polymer membranes. In general, the two main proton transport mechanisms are hopping (or Grotthus) and diffusion (or vehicular), which are both water-dependent. Water molecules in the membrane matrix can form a network of hydrogen bonds, thereby increasing the conduction of ions through the vehicular and Grotthuss mechanisms [29]. For fuel cell applications, the ion exchange capacity (IEC) of the polymer electrolyte membrane is an important factor because it indicates the number of ionic groups in the polymer matrix [30]. Hence, in the present work, ion exchange capacity and impedance were measured at room temperature (25 °C) for NdC10–NdC60. The highest measured ion exchange capacity was observed in NdC20 and proceeded to higher temperatures until 200 °C. The schematic diagram of the ion transport mechanism of phosphorylated NdC is shown in Figure 4.

**Figure 5:**
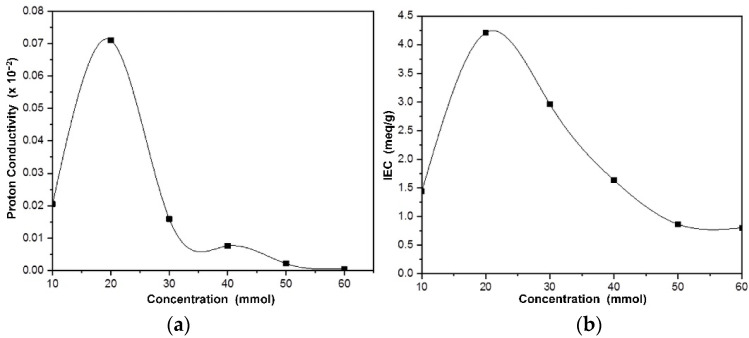
(**a**) Proton conductivity of phosphorylated NdC and (**b**) IEC of phosphorylated NdC.

As shown in Figure 5a, the addition of phosphoric acid into the NdC membrane improved the conductivity. NdC20 had the highest conductivity of 7.1 × 10^−2^ S cm^−1^ at room temperature. This finding correlates with the highest ion exchange capacity of NdC20. In Figure 5b, the ion exchange capacity represents the number of active sites or functional groups responsible for ion exchange in polymer electrolyte membranes. Hence, ion exchange capacity is a good indicator of ion availability, affects the ionic transfer, particularly in the Grotthuss mechanism, and is related to proton conduction. Table 2 shows the proton conductivity of NdC20 at various temperatures (25–200 °C). The proton conductivity significantly increased with temperature up to 80 °C and started to decrease sharply after 80 °C. The increasing hydronium ion (H_3_O^+^) transport is dominated by the support of the Grotthuss mechanism at high temperatures. However, high temperatures can decrease the humidity, lead to the disconnection of ion channels and reduce the conductivity of NdC20 at temperatures above 80 °C. Therefore, the role of water in the PEMFC system is very important because it acts as a bridge between conductive sites [31]. 

**Figure 6:**
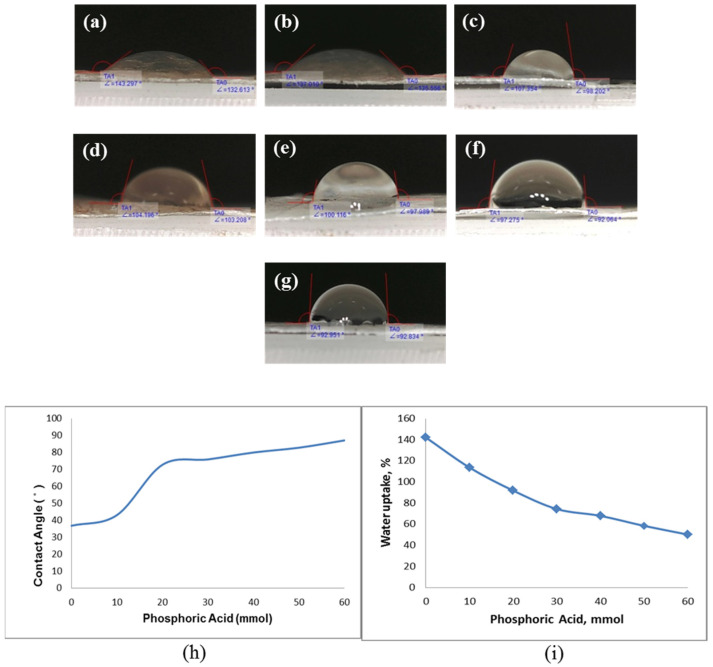
Contact angle of pure NdC and phosphorylated NdC: (**a**) pure NdC, (**b**) NdC10, (**c**) NdC20, (**d**) NdC30, (**e**) NdC40, (**f**) NdC50, and (**g**) NdC60; (**h**) effect of phosphoric acid treatment on contact angle of phosphorylated NdC; and (**i**) effect of phosphoric acid on water uptake of phosphorylated NdC.

Meanwhile, BC has a super hydrophilic characteristic because of the presence of various hydroxyl groups on the surface of polymer membranes. The highly hydrophilic nature of BC is the key to allowing proton transport across the membrane. Sufficient humidification of the membrane is important to achieve the desired proton conductivity and PEMFC performance. The high water content of composite membranes is the main reason for the high ionic conductivity. The presence of hydrophilic groups and the hydrogen bonding between water molecules and acids influence polymer proton conductivity. The ability of the electrolyte membrane to absorb water determines its ability to transport protons. The water contact angle is a critical parameter in determining the hydrophilicity of the membrane surface [32]. In the present study, the NdC membrane has high hydrophilicity, leading to a high degree of membrane swelling and low membrane performance. Figure 6 shows the effect of phosphorylation on contact angle. The introduction of phosphate groups into the NdC membrane decreases the ability of the membrane to absorb much water, causing the fragility of the phosphorylated NdC membrane. Crosslinked chains can be formed at high phosphoric acid concentrations because phosphoric acid acts as a crosslinker for NdC membranes and other polymers. Figure 6h shows that increasing the concentration of phosphoric acid results in a higher contact angle. Figure 6i shows that increasing the concentration of phosphoric acid in NdC reduces the hydrophilicity of the membrane because phosphorylation replaces the hydroxyl groups with less hydrophilic ones. Hence, the hydrophilicity of the phosphorylated NdC membranes decreases with phosphoric acid concentration. 

### 3.4. DMA of Phosphorylated NdC

Mechanical properties are strongly influenced by mesh size and fiber diameter. The mechanical properties of the NdC membranes were evaluated using Dynamic Mechanical Analysis (DMA). This technique is sensitive and provides data on polymer bulk properties, thermal transitions, and other minor phase or structural changes [33]. DMA test was conducted on the stretching test model. The results showed that the membrane storage moduli could represent Young’s moduli and provide information on the mechanical properties and glass transition of the membrane with high degrees of crystallinity or cross-linking. The tan δ and loss modulus peaks were represented using the glass transition temperature, with the tan δ peak occurring at a higher temperature than the loss modulus. Tan δ is well-known for being a good limit of the leather-like midpoint between the glassy and rubbery states. The storage modulus limits the recoverable stored strain energy, and the loss modulus limits the energy consumed and lost as heat [34,35]. 

**Figure 7:**
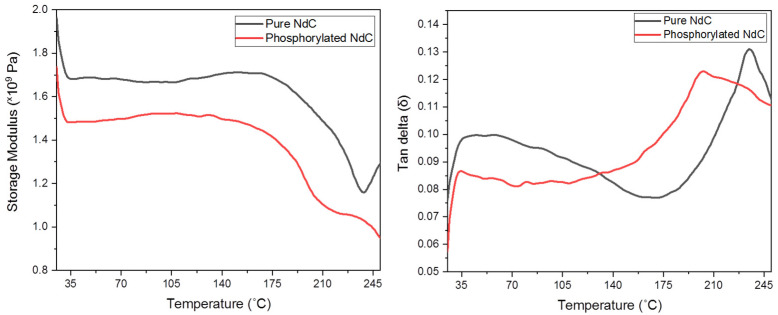
Tan δ and storage modulus of pure NdC and phosphorylated NdC20 membrane.

In general, DMA was used to determine the effect of the exposure of microcomposites to elevated temperatures on the stiffness of the polymer [36]. Figure 7 shows the plot of the tan δ and storage modulus versus the temperature of pure NdC and phosphorylated NdC20 membrane. Tg occurred within 180–235 °C for all membranes. NdC20 was selected due to its high performance in proton conductivity. The Tg values are 234.25 °C for pure Ndc and decrease to around 190 °C for the phosphorylated NdC20. The range of Tg peak heights of pure NdC and phosphorylated NdC20 membranes is approximately 0.12–0.13 based on the tan δ curve in Figure 7. The range of Tg peaks mentioned proved that all the membranes were in the crystalline phase, consistent with the XRD analysis in Section 3.2.

The storage modulus of pure NdC is 1.67 × 10^9^ at 35 °C; it slightly decreased at 75 °C, started to slightly increase at 107–175 °C, and then decreased again until above 210 °C. The phosphorylated NdC membranes decreased from 1.67 × 10^9^ to 1.48 × 10^9^ in NdC20 at 35 °C. The storage modulus values demonstrated that the phosphorylated NdC membrane has good mechanical stability and could be a promising membrane material for fuel cell applications.

### 3.5. MEA and Single Cell Performance of Phosphorylated NdC

**Figure 8:**
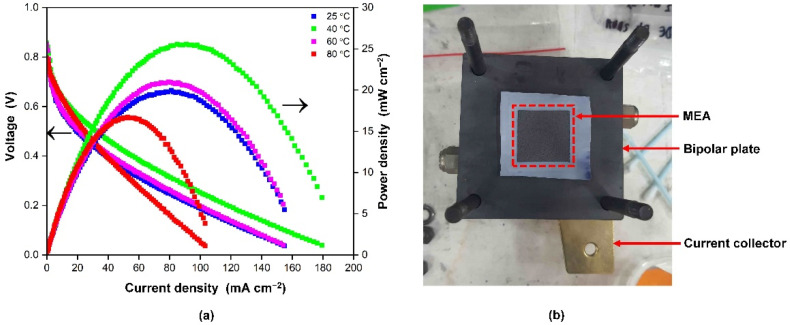
(**a**) Single-cell performance of phosphorylated NdC with different temperatures, (**b**) MEA assembly in PEMFC single-cell set-up.

In consideration of proton conductivity and IEC results discussed in Section 3.3, the addition of phosphoric acid into the NdC membrane improved the conductivity of the membrane. The highest conductivity was obtained in NdC20, with a value of 7.9 × 10^−2^ S cm^−1^ at 80 °C. NdC20 membrane was selected for fabrication of membrane electrode assembly and PEM fuel cell performance test. Figure 8a shows the polarization curves and power density profiles of the fuel cell at various operating temperatures from 25 to 80 °C. Figure 8b displays MEA assembly in the PEMFC single-cell set-up. The initial decline in the polarization curve was caused by a loss of the active catalyst surface area and a reduction in the electrical connectivity of the catalyst support structure at the electrode [37]. In the present study, phosphorylated NdC20-based MEA for single-cell performance was lower than the single-cell performance of commercial membrane fuel cells; however, the value of proton conductivity was higher than modified bacterial cellulose elsewhere [38]. 

Furthermore, these curves indicate that the fuel cell performance was improved to 25 mW cm^−2^ with increasing temperature from 25 °C to 40 °C and started to decrease at 60 °C. The increase in fuel cell performance between 25 °C to 40 °C can be clarified by the increase in the gas diffusivity and membrane conductivity at higher temperatures. Water is easily condensed at a lower temperature; water flooding may degenerate gas diffusivity in the catalyst and gas diffusion layer. The gas diffusivity of the fuel cell was improved with increasing fuel cell temperature; therefore, the performance increased at a higher temperature. Nonetheless, at 60 °C, the membrane conductivity decreased as the relative humidity of the reactant gases, and the water content in the membrane decreased. As a result, when the temperature was raised to 80 °C, the fuel cell performance suffered. The rate of water evaporation increased as the temperature increased. When the temperature reached a critical point where the amount of water evaporated exceeded the amount of water produced, the membrane began to dry out. As the membranes dried out, the resistance increased, reducing the current and water production [39].

Herein, the efficiency of a fuel cell is determined by the efficiency of the electrode, catalyst activity, great ionic conductivity, and IEC, and the membrane should have high ionic conductivity to achieve higher fuel cell performance [40], although the single-cell performance of the NdC20 membrane was lower than commercial membrane fuel cells, the value of proton conductivity was higher than modified bacterial cellulose elsewhere [39] indicating the promising membrane to be used as a proton exchange membrane which more environmentally friendly, and inexpensive alternative membrane for fuel cell application.

## 4. Conclusions

The phosphorylated NdC (nata de cassava) membranes were successfully modified and doped with phosphoric acid to significantly improve the proton conductivity (maximum conductivity up to 7.9 × 10^−2^ S cm^−1^ at temperature 80 °C), IEC, mechanical properties, and contact angle. High power density for single-cell performance test at about 25 mW cm^−2^ was obtained for MEA fabricated using NdC20 membrane with 60 wt% Pt/C loading of 0.5 mg cm^−2^ for both anode and cathode. In summary, the phosphorylation process on the NdC membrane has successfully improved its properties; hence this membrane has the potential to be used as a proton exchange membrane in fuel cell applications.

## Figures and Tables

**Table 1:** Fiber size (diameter) of phosphorylated Nata de Cassava membranes.

NdC Membranes	Fiber Size (nm)
Pure NdC	67.5
10 mmol H_3_PO_4_	69.1
20 mmol H_3_PO_4_	93.0
30 mmol H_3_PO_4_	106.5
40 mmol H_3_PO_4_	117.2
50 mmol H_3_PO_4_	120.5
60 mmol H_3_PO_4_	415.4

**Table 2:** Proton conductivity of NdC20 at various temperatures (25–200 °C).

Temperature (°C)	Conductivity (S cm^−1^)
25	7.1 × 10^−2^
40	7.3 × 10^−2^
80	7.9 × 10^−2^
120	6.4 × 10^−4^
200	8.4 × 10^−^^6^

## Data Availability

Not applicable.

## References

[1] Ling S., Chen W., Fan Y., Zheng K., Jin K., Yu H., Buehler M.J., Kaplan D.L. (2018). Biopolymer Nanofibrils: Structure, Modeling, Preparation, and Applications. Prog. Polym. Sci..

[2] Zaman A., Huang F., Jiang M., Wei W., Zhou Z. (2020). Preparation, Properties, and Applications of Natural Cellulosic Aerogels: A Review. Energy Built Environ..

[3] Panaitescu D.M., Lupescu I., Frone A.N., Chiulan I., Nicolae C.A., Tofan V., Stefaniu A., Somoghi R., Trusca R. (2017). Medium Chain-Length Polyhydroxyalkanoate Copolymer Modified by Bacterial Cellulose for Medical Devices. Biomacromolecules.

[4] Klemm D., Heublein B., Fink H.P., Bohn A. (2005). Cellulose: Fascinating Biopolymer and Sustainable Raw Material. Angew. Chem.-Int. Ed..

[5] Mohite B.V., Patil S.V. (2014). A Novel Biomaterial: Bacterial Cellulose & Its New Era Applications. Biotechnol. Appl. Biochem.

[6] Wong C.Y., Wong W.Y., Loh K.S., Khalid M., Wan Daud W.R., Lim K.L., Walvekar R. (2020). Influences of Crosslinked Carboxylic Acid Monomers on the Proton Conduction Characteristics of Chitosan/SPVA Composite Membranes. Polymer (Guildf.).

[7] Brown J.J. (1886). XLIII.—On an acetic ferment which forms cellulose. J. Chem. Soc. Trans..

[8] Mohite B.V., Patil S.V. (2014). Physical, Structural, Mechanical and Thermal Characterization of Bacterial Cellulose by G. Hansenii NCIM 2529. Carbohydr. Polym..

[9] Wang H., Zhu E., Yang J., Zhou P., Sun D., Tang W. (2012). Bacterial Cellulose Nano Fiber-Supported Polyaniline Nanocomposites with Flake-Shaped Morphology as Supercapacitor Electrodes. J. Phys. Chem. C.

[10] Shao W., Wu J., Liu H., Ye S., Jiang L., Liu X. (2017). Novel Bioactive Surface Functionalization of Bacterial Cellulose Membrane. Carbohydr. Polym..

[11] Gao M., Li J., Bao Z., Hu M., Nian R., Feng D., An D., Li X., Xian M., Zhang H. (2019). A Natural in Situ Fabrication Method of Functional Bacterial Cellulose Using a Microorganism. Nat. Commun..

[12] Clasen C., Sultanova B., Wilhelms T., Heisig P., Kulicke W.M. (2006). Effects of Different Drying Processes on the Material Properties of Bacterial Cellulose Membranes. Macromol. Symp..

[13] Shah N., Ul-Islam M., Khattak W.A., Park J.K. (2013). Overview of Bacterial Cellulose Composites: A Multipurpose Advanced Material. Carbohydr. Polym..

[14] Ye J., Guo L., Zheng S., Feng Y., Zhang T., Yang Z., Yuan Q., Shen G., Zhang Z. (2019). Synthesis of Bacterial Cellulose Based SnO2-Pyy Nanocomposites as Potential Flexible, Highly Conductive Material. Mater. Lett..

[15] Luo H., Xie J., Xiong L., Zhu Y., Yang Z., Wan Y. (2019). Fabrication of Flexible, Ultra-Strong, and Highly Conductive Bacterial Cellulose-Based Paper by Engineering Dispersion of Graphene Nanosheets. Compos. Part B Eng..

[16] Hosseini H., Teymouri M., Saboor S., Khalili A., Goodarzi V., Poudineh Hajipoor F., Khonakdar H.A., Shojaei S., Asefnejad A., Bagheri H. (2019). Challenge between Sequence Presences of Conductive Additives on Flexibility, Dielectric and Supercapacitance Behaviors of Nanofibrillated Template of Bacterial Cellulose Aerogels. Eur. Polym. J..

[17] Sheng N., Chen S., Yao J., Guan F., Zhang M., Wang B., Wu Z., Ji P., Wang H. (2019). Polypyrrole@TEMPO-Oxidized Bacterial Cellulose/Reduced Graphene Oxide Macrofibers for Flexible All-Solid-State Supercapacitors. Chem. Eng. J..

[18] Lin C.W., Chen S.W. (2012). Modification and Characterization of Bacterial Cellulose Biopolymer as Proton Conducting Membrane. Proc. World Acad. Sci. Eng. Technol. (No. 65).

[19] Vilela C., Silva A.C.Q., Domingues E.M., Gonçalves G., Martins M.A., Figueiredo F.M.L., Santos S.A.O., Freire C.S.R. (2020). Conductive Polysaccharides-Based Proton-Exchange Membranes for Fuel Cell Applications: The Case of Bacterial Cellulose and Fucoidan. Carbohydr. Polym..

[20] Gadim T.D.O., Loureiro F.J.A., Vilela C., Rosero-Navarro N., Silvestre A.J.D., Freire C.S.R., Figueiredo F.M.L. (2017). Protonic Conductivity and Fuel Cell Tests of Nanocomposite Membranes Based on Bacterial Cellulose. Electrochim. Acta.

[21] Naumi F., Hendrana S., Fadlinatin N., Natanael C.L., Iman R., Lucia I., Sunit H. (2018). Polymer Electrolyte Membrane Fuel Cell Based on Sulfonated Polystyrene and Phosphoric Acid with Biocellulose as a Matrix Riset Unggulan LIPI View Project Development Membrane for Fuelcell View Project Polymer Electrolyte Membrane Fuel Cell Based on Sulfonated Polystyrene and Phosphoric Acid with Biocellulose as a Matrix. Res. J. Chem. Environ..

[22] Sari A.K., Majlan E.H., Loh K.S., Wong W.Y., Alva S., Khaerudini D.S., Yunus R.M. (2022). Effect of Acid Treatments on Thermal Properties of Bacterial Cellulose Produced from Cassava Liquid Waste. Mater. Today Proc..

[23] Menéndez J.C. (2006). Microwave Assisted Organic Synthesis. Synthesis (Stuttg.).

[24] Zeng M., Laromaine A., Roig A. (2014). Bacterial Cellulose Films: Influence of Bacterial Strain and Drying Route on Film Properties. Cellulose.

[25] Jiang G.P., Zhang J., Qiao J.L., Jiang Y.M., Zarrin H., Chen Z., Hong F. (2015). Bacterial Nanocellulose/Nafion Composite Membranes for Low Temperature Polymer Electrolyte Fuel Cells. J. Power Sources.

[26] Yue L., Xie Y., Zheng Y., He W., Guo S., Sun Y., Zhang T., Liu S. (2017). Sulfonated Bacterial Cellulose/Polyaniline Composite Membrane for use as Gel Polymer Electrolyte. Compos. Sci. Technol..

[27] Hadid M., Noukrati H., Ben youcef H., Barroug A., Sehaqui H. (2021). Phosphorylated Cellulose for Water Purification: A Promising Material with Outstanding Adsorption Capacity towards Methylene Blue. Cellulose.

[28] Barud H.S., Ribeiro C.A., Crespi M.S., Martines M.A.U., Dexpert-Ghys J., Marques R.F.C., Messaddeq Y., Ribeiro S.J.L. (2007). Thermal Characterization of Bacterial Cellulose-Phosphate Composite Membranes. J. Therm. Anal. Calorim..

[29] Wu H., Hou W., Wang J., Xiao L., Jiang Z. (2010). Preparation and Properties of Hybrid Direct Methanol Fuel Cell Membranes by Embedding Organophosphorylated Titania Submicrospheres into a Chitosan Polymer Matrix. J. Power Sources.

[30] Rosli N.A.H., Loh K.S., Wong W.Y., Lee T.K., Ahmad A. (2021). Hybrid Composite Membrane of Phosphorylated Chitosan/Poly (Vinyl Alcohol)/Silica as a Proton Exchange Membrane. Membranes.

[31] Wong C.Y., Wong W.Y., Ramya K., Khalid M., Loh K.S., Daud W.R.W., Lim K.L., Walvekar R., Kadhum A.A.H. (2019). Additives in Proton Exchange Membranes for Low- and High-Temperature Fuel Cell Applications: A Review. Int. J. Hydrogen Energy.

[32] Branco C.M., El-kharouf A., Du S. (2017). Materials for Polymer Electrolyte Membrane Fuel Cells (PEMFCs): Electrolyte Membrane, Gas Diffusion Layers, and Bipolar Plates.

[33] Rosli N.A.H., Loh K.S., Wong W.Y., Lee T.K., Ahmad A. (2022). Phosphorylated Chitosan/Poly(Vinyl Alcohol) Based Proton Exchange Membranes Modified with Propylammonium Nitrate Ionic Liquid and Silica Filler for Fuel Cell Applications. Int. J. Hydrogen Energy.

[34] Menard K.P. (2004). Dynamic Mechanical Analysis. Encycl. Polym. Sci. Technol..

[35] Menard K.P., Bilyeu B.W. (2008). Dynamic Mechanical Analysis of Polymers and Rubbers. Encycl. Anal. Chem..

[36] Sukyai P., Sriroth K., Lee B.H., Kim H.J. (2012). The Effect of Bacterial Cellulose on the Mechanical and Thermal Expansion Properties of Kenaf/Polylactic Acid Composites. Appl. Mech. Mater..

[37] Antolini E. (2003). Formation, Microstructural Characteristics and Stability of Carbon Supported Platinum Catalysts for Low Temperature Fuel Cells. J. Mater. Sci..

[38] Radiman C.L., Rifathin A. (2013). Preparation of Phosphorylated Nata-de-Coco for Polymer Electrolyte Membrane Applications. J. Appl. Polym. Sci..

[39] Selyanchyn O., Selyanchyn R., Lyth S.M. (2020). A Review of Proton Conductivity in Cellulosic Materials. Front. Energy Res..

[40] Vijayalekshmi V., Khastgir D. (2017). Eco-Friendly Methanesulfonic Acid and Sodium Salt of Dodecylbenzene Sulfonic Acid Doped Cross-Linked Chitosan Based Green Polymer Electrolyte Membranes for Fuel Cell Applications. J. Memb. Sci..

